# The Impact of Storage Conditions on the Gas-Forming Tendency of Moulds and Cores Made with Resole-Type Phenol Formaldehyde Resin

**DOI:** 10.3390/ma18214832

**Published:** 2025-10-22

**Authors:** Artur Bobrowski, Faustyna Woźniak, Sylwia Żymankowska-Kumon, Hubert Ziętal, Kacper Januszek, Beata Grabowska

**Affiliations:** Faculty of Foundry Engineering, AGH University of Krakow, Reymonta 23, 30059 Krakow, Poland; wozfau@agh.edu.pl (F.W.); szk@agh.edu.pl (S.Ż.-K.); hzietal@student.agh.edu.pl (H.Z.); januszek@student.agh.edu.pl (K.J.); beata.grabowska@agh.edu.pl (B.G.)

**Keywords:** resole phenolic resin, gas emissions in foundry, BTEX compounds, mould and core storage conditions, alkaline phenolic no-bake (APNB)

## Abstract

The article presents the results of a study aimed at determining the impact of storage conditions on the gas-forming tendency of standard samples (cores) made from moulding sand using a two-component binder based on resole-type phenolic resin, cured with a dedicated ester mixture. The objective of the research was to determine the total volume of gases released as a result of contact between the cores or moulds and the high temperature of molten casting alloys, as well as the rate of gas release, which can influence the tendency for gas-related casting defects. Additionally, the influence of storage conditions on the gas-forming tendency of the samples was evaluated in terms of their environmental and occupational health impact, based on the emission of BTEX compounds (benzene, toluene, ethylbenzene, and xylenes), which serve as key indicators of the harmfulness of moulding and core sands to the surrounding environment.

## 1. Introduction

Foundry engineering is a highly significant branch of industry, playing a crucial role in the development of many other industrial sectors, particularly the automotive, machinery, aerospace, and energy industries. The growing demands of modern manufacturing require continuous improvement in the field of materials engineering used in foundry processes—not only in terms of the quality of the casting alloy, which must meet specific requirements regarding composition, crystallization behavior, and mechanical properties, but also in the design and preparation of the moulds that define the intended shape of the casting. In this context, properly selected materials for moulds and cores are of particular importance—both the ceramic matrix materials that form the base of moulding and core sands, and the binders that hold the grain structure together. These binders largely determine the technological properties of the moulding and core sands, which must exhibit adequate gas permeability, flowability, and compactability. The type and amount of binder used also directly affect the mechanical properties of the moulds and cores, such as compressive, tensile, and bending strength, as well as high-temperature resistance and gas-forming tendency [1,2]. The latter is especially important, as excessive gas evolution can lead to gas-related defects in castings, often disqualifying them from use due to issues such as porosity or leakage [3,4]. Increasing environmental regulations and the drive to improve working conditions in foundries are also pushing the industry toward the use of low-emission materials, particularly in relation to the emission of hydrocarbons [5,6]. In the context of moulding and core sands used in foundry processes, the emission of BTEX compounds (benzene, toluene, ethylbenzene, and xylenes) is commonly used as a measure of their environmental and occupational health impact. A substantial body of literature exists concerning the evaluation of gas-forming tendencies in moulding and core sands, with significant contributions made by the co-authors of this paper [7,8]. Most commercially available binders were tested. The effects of the type of organic resin and hardener used in the preparation of molding sands (furfuryl, alkyd, and phenol formaldehyde resins) and cores were analyzed. Gas emission studies were also conducted for systems using inorganic binders (such as water glass and geopolymers) and biopolymer-based binders. Since the use of reclaimed sand—produced through mechanical and/or thermal processing of spent molding sands—is a common practice in foundries, gas emission studies were carried out with varying proportions of reclaimed sand derived from furfuryl resin molding sand. This study investigated the effect of reclaimed sand content on the total volume of released gases and on the emission of BTEX compounds. However, from a technological standpoint, there is a lack of data regarding the impact of storage conditions on the gas-forming tendency of moulds and cores [9,10]. In practice, cores are often produced in advance and stored in warehouses or designated areas of the foundry. As a result, they are exposed to varying climatic conditions, changes in temperature and humidity, until they are eventually placed into the mould cavity and poured with molten metal. Although their mechanical strength may remain sufficient for safe handling, foundries often lack information on potential absorption (in the case of hygroscopic binders) and/or moisture adsorption on the surface of the cores or moulds. Due to emission restrictions, the APNB (Alkaline Phenolic No-Bake) technology is increasingly replacing widely used binders based on furfuryl alcohol [1,11]. The phenol formaldehyde resole resin used in this study is intended for the Alphaset process, also known as APNB. This is a two-component system in which a dedicated dimethyl ester mixture acts as the curing agent. One of the key advantages of this technology is that no heat or gas purging is required to initiate curing of the moulding or core sand. The binder consists of a phenol formaldehyde resole resin in an aqueous alkaline medium (NaOH, KOH). During the initial curing phase, partial polymerization of the resole chains occurs, accompanied by the removal of metal ions (Na^+^, K^+^) through ester hydrolysis in the alkaline environment. The ester mixture, tailored to the specific resin, varies in hydrolysis rate, which affects acid release and thus controls curing speed and the working time of the sand—the so-called “bench life” [5,12]. Typically, the binder content in this system ranges from 1.2% to 1.8% relative to the mass of the sand matrix, depending on the size of the mould and its purpose (moulding sand or core sand) [13]. Another benefit of the APNB system is its thermoplastic deformation behavior upon contact with molten metal: the cured bonds temporarily loosen, followed by a secondary hardening phase. This mechanism compensates for the reversible volume change in quartz sand at 573 °C caused by polymorphic transformation, preventing cracking and reducing casting defects such as veining [14,15,16]. Spent moulding sands from the APNB process are suitable for reclamation—the recovery of the grain matrix through mechanical, thermal, or combined treatments. Mechanical reclamation alone allows for approximately 70% sand recovery, while combined mechanical-thermal methods can achieve up to 95% recovery rates [5,8,14,17,18,19]. The APNB system contains no sulfur or nitrogen, making it highly versatile and suitable for casting all types of metals, including cast iron and steel [5,20]. It has been documented that when metal is poured into moulds bonded with APNB resin, the bonds temporarily relax before re-hardening, preventing cracking and minimizing veining defects. Castings produced using this method exhibit high dimensional accuracy [14,15].

The objective of this study is to evaluate the effect of storage conditions on the total amount and rate of gas emission, as well as the environmental impact of these gases—particularly BTEX compounds—from samples made using resole-type phenol formaldehyde resin cured with an ester mixture. The study focuses not only on the storage conditions themselves but also on the time interval between core production and their placement in the mould cavity and pouring with molten metal—a critical factor in foundry operations.

## 2. Research Materials and Methodology

The following materials were used to prepare the samples:

- Grain matrix, quartz sand (parameters in Table 1) DBCargo, Szczakowa, Poland;
- Resol-type phenol formaldehyde resin [Prec-Odlew, Skawina, Poland];
- Hardener, a mixture of dimethyl esters [Prec-Odlew, Skawina, Poland].

The moulding/core sand was prepared according to the following procedure:

- Grain matrix 100 parts by mass;
- Resin 1.5 parts by weight relative to the grain matrix;
- Hardener 25% relative to the resin.

**Figure 1:**
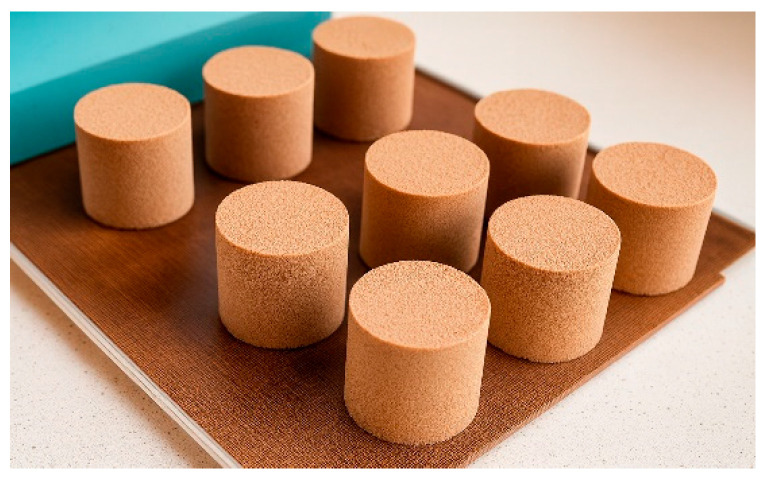
Standard cylindrical samples.

The weighed components were mixed in a rotor-type mixer. First, the hardener was added and mixed with quartz sand for 30 s, followed by the addition of the resin, with an additional mixing time of 30 s. Standard cylindrical test samples with dimensions of Φ50 × 50 mm were prepared from the moulding mixture using vibratory compaction, employing a LUZ-2 e vibratory compaction device for moulding sands [21] (manufactured by Multiserw Morek). The samples (Figure 1) were then set aside for complete curing. After 6 h (except for the set stored in the laboratory for 3 h), the samples were subjected to the following storage conditions:

- 24 h in a glass desiccator;
- 3 h under laboratory conditions (Temperature [T] = 20–21 °C, relative humidity [RH] = 45–50%);
- 24 h under laboratory conditions (Temperature [T] = 20–21 °C, relative humidity [RH] = 45–50%);
- 7 days under laboratory conditions (Temperature [T] = 20–21 °C, relative humidity [RH] = 45–50%);
- 24 h in a climate chamber: temperature T = 35 °C, relative humidity RH = 70%.

**Figure 2:**
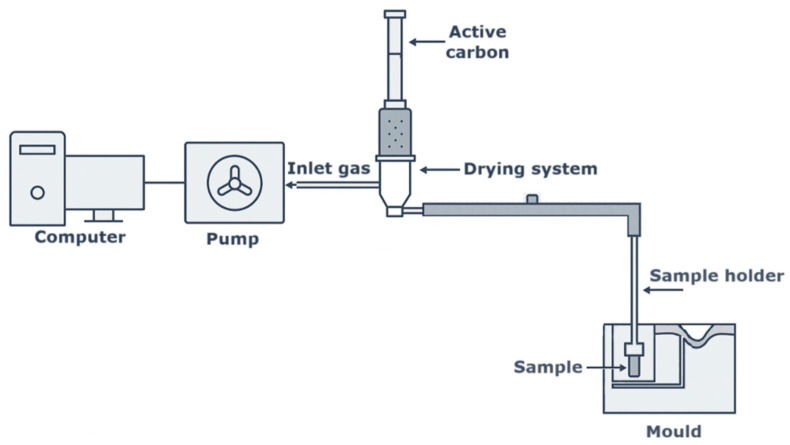
Station for testing the intensity of gas emissions and harmfulness ([22]).

**Figure 3:**
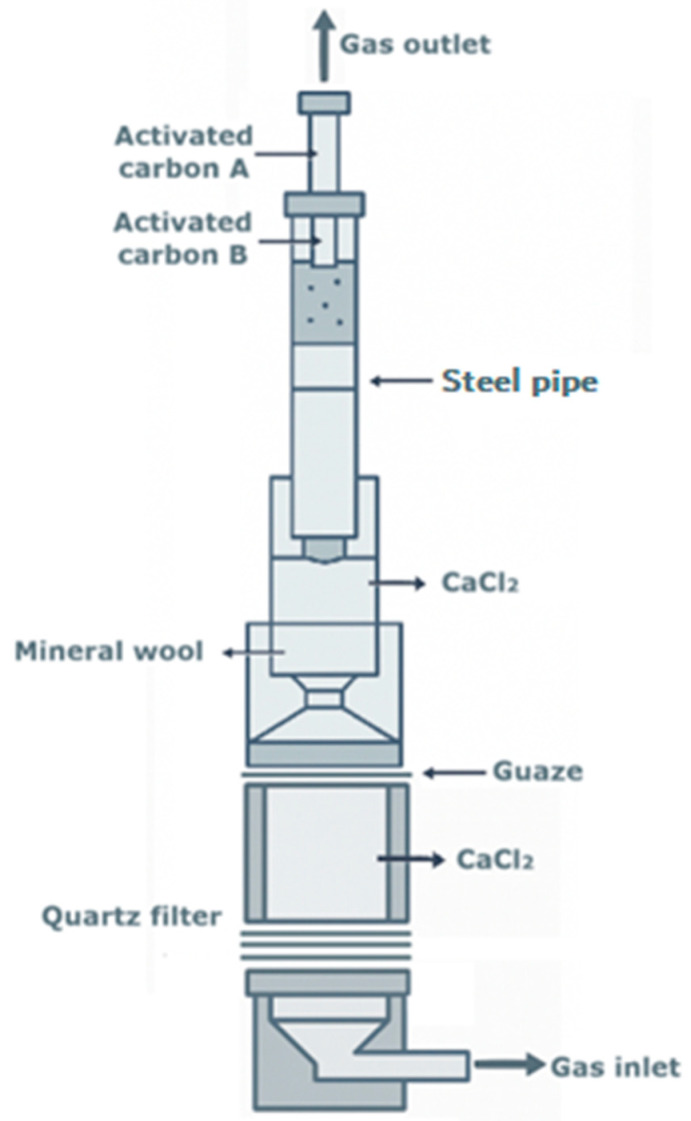
Capsule with activated carbon bed for collecting BTEX group compounds ([22]).

Measurements of gas volume and emission kinetics, along with the analysis of BTEX group compounds (benzene, toluene, ethylbenzene, xylenes), were conducted using a patented testing stand designed to assess the emission intensity and harmfulness of gases released from technological materials used in foundry and metallurgical processes [22] (Figure 2). For each sample, two repeatable measurements were performed. A previously weighed cylindrical sample (Ø50 × 50 mm) was placed in a steel “bell” and then inserted into a pre-prepared mould cavity made of bentonite-bonded moulding sand. The sample was then poured with molten cast iron at a temperature of 1350 °C. The total mass of molten metal used in the mould was approximately 9 kg. As a result of the thermal impact of the molten alloy, thermal degradation of the binder in the moulding/core sample occurred. The resulting gases were directed into a collection capsule (Figure 2), which includes a gas drying unit. The gas stream was then passed through a bed of activated carbon for adsorption (Figure 3) and subsequently routed through a calibrated system of tubes to a peristaltic pump connected to a gas volume recorder. Qualitative and quantitative analyses of the emitted gases were performed using gas chromatography with a flame ionization detector (FID, Thermo Scientific, Waltham, MA, USA). The activated carbon sorbent (charcoal activated for gas chromatography, particle size 0.5–1 mm; 18–35 mesh American Society for Testing and Materials ASTM; CAS 7440-44-0, molar mass 12.01 g/mol), containing adsorbed organic compounds, was subjected to extraction with diethyl ether. The solvent volume required for the complete desorption of BTEX compounds from the activated carbon column was determined as follows: 40 mL of diethyl ether for the primary bed, applied in four successive aliquots of 10 mL, and 10 mL of diethyl ether for the backup bed [23]. Identification of BTEX compounds was carried out using a Trace GC Ultra gas chromatograph (Thermo Scientific, Thermo Fisher Scientific Inc., Milan, Italy) equipped with a capillary column (Rtx^®^-5 MS, fused silica, low-polarity phase, cross-linked 5% diphenyl/95% dimethylpolysiloxane; Restek™, Bellefonte, PA, USA), 30 m in length and 0.25 mm in internal diameter [24]. Chromatographic separation was conducted under the following conditions: an initial temperature of 40 °C maintained for 3 min; a linear increase to 100 °C at a rate of 3 °C·min^−1^ with a 3 min hold; followed by heating to 250 °C at a rate of 20 °C·min^−1^ and a 3 min hold. The carrier gas was helium, with a column flow rate of 1 mL·min^−1^, and the injector operated in SPLIT (volume ratio of the two streams) mode with a 1:30 split ratio. The analytical protocol was based on standardized procedures described in the AFS Mold & Core Test Handbook (American Foundry Society) and the international standards ISO 16017 and EN 13649 [22].

## 3. Results

To facilitate the interpretation of the results, the sample designations (S1–S5) and their corresponding storage conditions are summarized in Table 2. All curves presented in the figures represent mean ± SD values obtained from three independent measurements (n = 3).

**Figure 4:**
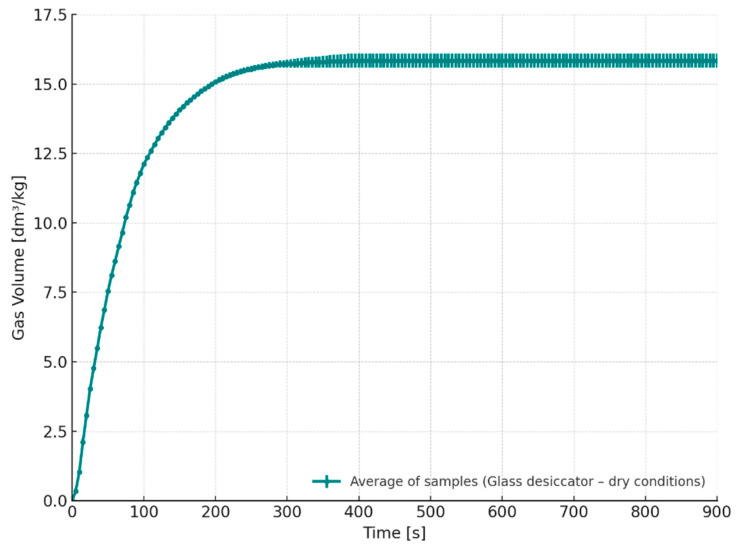
Released gas volume as a function of time for cores stored under dry conditions (S1–24 h).

Figure 4 presents the relationship between the volume of gases emitted over time from core samples stored for 24 h in a desiccator under dry conditions. The presented curve corresponds to the average values obtained from three independent measurements (n = 3). The total volume of emitted gases reached approximately 15.7 dm^3^/kg.

**Figure 5:**
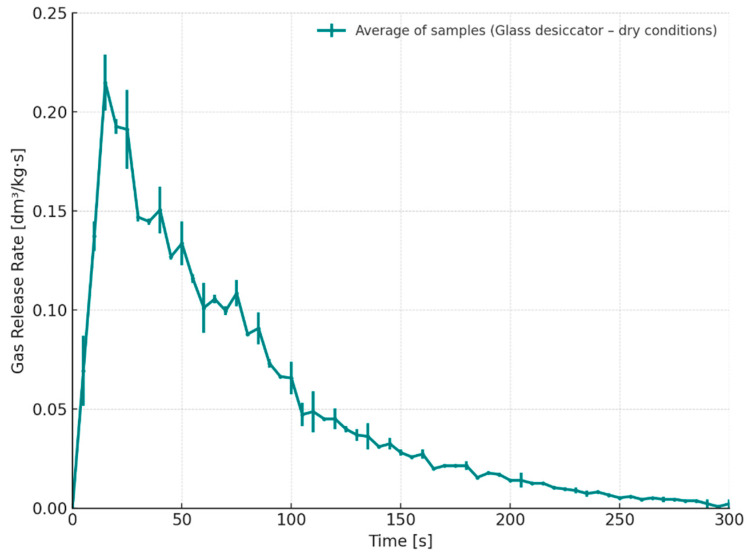
Gas release rate as a function of time for cores stored in a glass desiccator under dry conditions (S1–24 h).

Figure 5 illustrates the gas emission rate profiles for the tested core samples following 24 h of storage in a desiccator. The emission rate was recorded continuously from the moment of contact between the molten cast iron and the mould cavity. Both samples demonstrated similar kinetic profiles, indicating a high degree of repeatability in gas release behavior under controlled storage conditions. The most intensive gas evolution occurred within the first 20–40 s after metal pouring, reaching a peak emission rate of approximately 0.23 dm^3^/(kg·s). This sharp increase is associated with the initial thermal decomposition of the resin binder due to the rapid temperature rise induced by the molten alloy (1350 °C). Following the peak, the emission rate gradually decreased, reflecting the progressive exhaustion of volatile components within the binder system. The early onset and intensity of gas evolution are of particular importance in foundry practice, as they directly influence the formation of gas-related casting defects. Rapid gas release in this time window can lead to pressure buildup in the mould cavity if not properly vented, increasing the risk of porosity or gas blows. Therefore, understanding this emission behavior is crucial for optimizing mould design, binder selection, and storage protocols.

**Figure 6:**
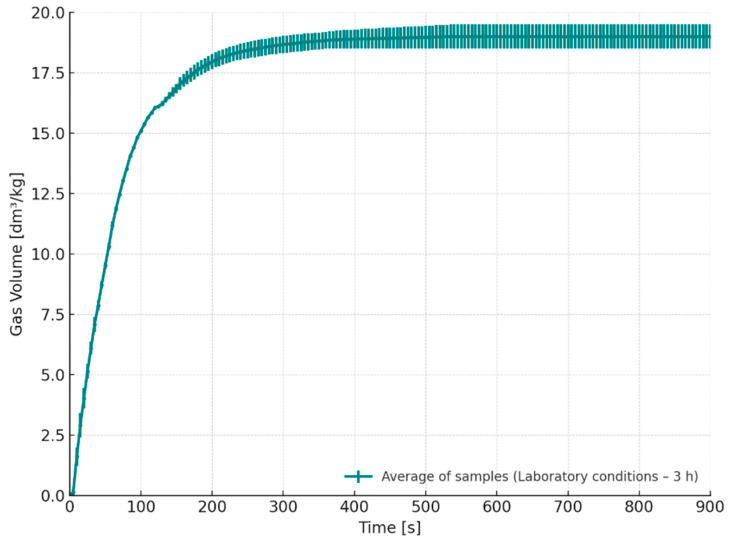
Released gas volume as a function of time for cores stored under laboratory conditions for 3 h (S2–3 h).

Figure 6 presents the gas emission volume as a function of time for ores stored under laboratory conditions for 3 h (S2). In contrast to the samples stored in a desiccator, the gas emission for this series was prolonged, with the most significant increase occurring up to approximately 400 s after contact with the high-temperature molten alloy. The total gas volume emitted reached approximately 19.0 dm^3^/kg, which is about 2 dm^3^/kg higher compared to the desiccator-stored samples, indicating a significantly greater gas evolution. Such an increase in gas release may considerably raise the risk of gas-related casting defects, particularly if the generated gases are not effectively removed from the mould cavity. The increased gas evolution is attributed to the shorter time interval between core fabrication and their exposure to molten metal, which likely resulted in incomplete curing of the binder, especially in the internal structure of the samples. These findings indicate that insufficiently cured cores can substantially contribute to defect formation during casting, due to excessive gas evolution under thermal stress. This underlines the importance of ensuring adequate curing time before storage or use, particularly when using resin-bonded sand systems. Delays in full polymer crosslinking, especially in larger or more thermally insulated cores, may result in residual binder decomposition during pouring, increasing porosity risk.

**Figure 7:**
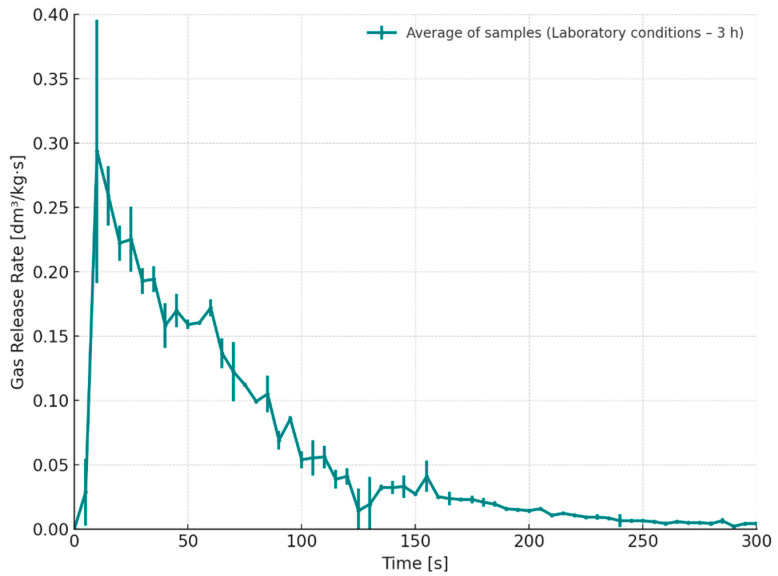
Gas release rate as a function of time for cores stored under laboratory conditions (S2–3 h).

Figure 7 presents the gas emission rate over time for cores stored under laboratory conditions for 3 h after preparation (S2). The results clearly indicate minor variations in the emission profile, which may stem from uneven distribution of the binder or varying degrees of curing throughout the sample. Compared with the desiccator-stored samples (S1), the gas emission in this series was more prolonged, with the peak occurring approximately 40–50 s after pouring molten cast iron—about 10 s later than in the case of dry-stored samples. The averaged peak emission rate reached approximately 0.38 dm^3^/(kg·s), indicating a higher intensity of gas evolution under ambient storage conditions. Such a sudden increase suggests localized regions within the core that responded more intensely to thermal degradation, likely due to insufficient or uneven curing. After the peak, the emission rate gradually decreased, reflecting the progressive exhaustion of volatile components within the binder system. Observed differences in the gas emission profiles suggest that the intensity and nature of gas release may be influenced by the storage conditions of the samples and the time elapsed between their preparation and pouring with molten metal, which may be related to the degree of binder curing in moulding or core sand.

**Figure 8:**
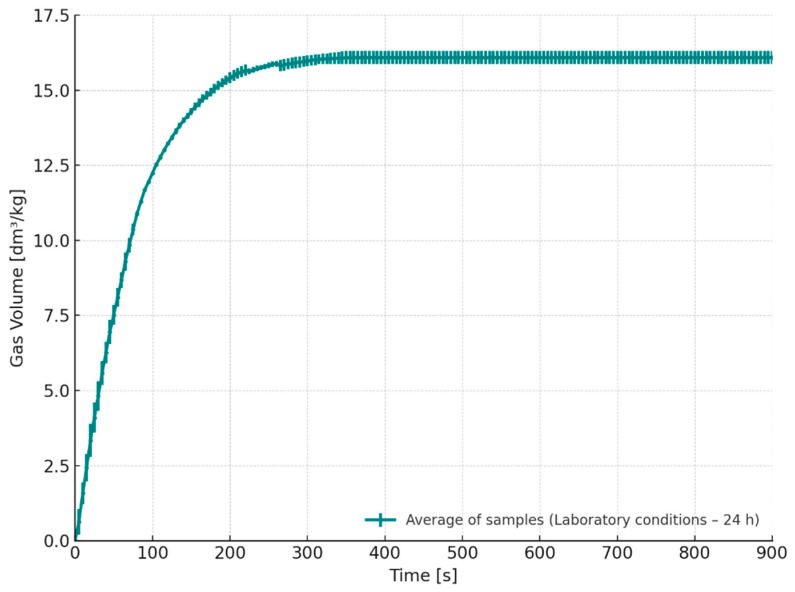
Released gas volume as a function of gas released over time for cores stored under laboratory conditions for 24 h (S3–24 h).

Figure 8 presents the gas emission curve over time for cores stored for 24 h under laboratory conditions (S3, T = 20–21 °C, RH = 45–50%). The results show that the total volume of emitted gases was comparable to that obtained for the desiccator-stored series (S1). This indicates that under moderate and stable humidity conditions, the binder system retained its thermal stability, and no significant increase in gas evolution was observed. Such behavior suggests that maintaining controlled climatic conditions during the storage of cores or moulds may help to prevent undesirable physicochemical changes in the binder, which could otherwise lead to increased gas release during pouring.

**Figure 9:**
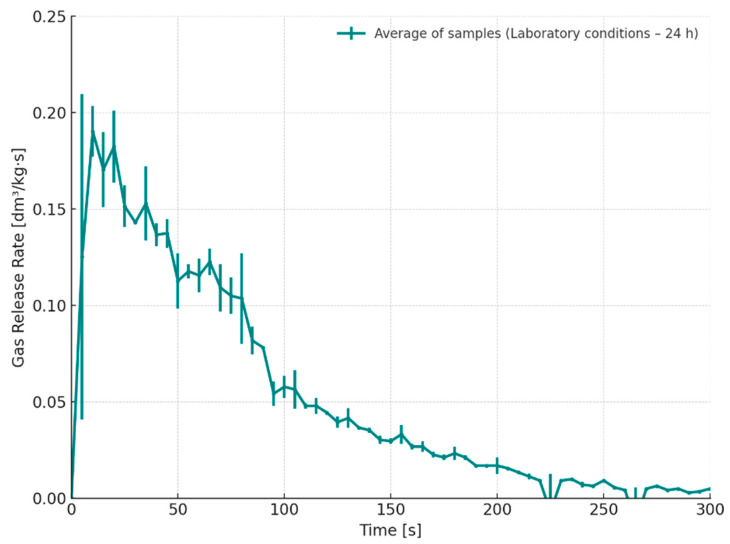
Gas release rate as a function of time for cores stored under laboratory conditions (S3–24 h).

Figure 9 illustrates the gas emission rate over time for cores stored for 24 h under laboratory conditions (S3, T = 20–21 °C, RH = 45–50%). The emission characteristics for this series closely resembled those of the desiccator-stored cores (S1) in terms of total gas volume and peak emission values. However, a slight shift in the onset of gas evolution was observed, occurring only a few seconds after contact with the molten alloy. This earlier initiation of gas release allowed the evolved gases to escape more gradually from the mould cavity, potentially reducing the risk of gas entrapment. Adequate venting during this initial stage of pouring may therefore help to minimize the formation of gas-related casting defects.

**Figure 10:**
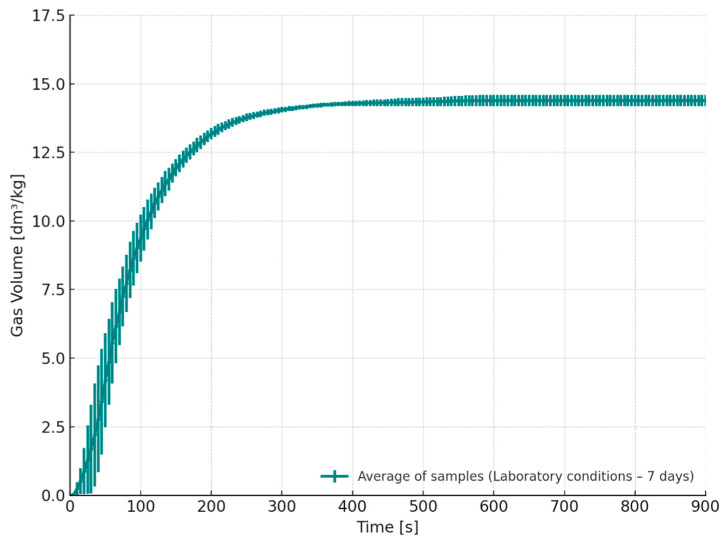
Released gas volume as a function of time for cores stored under laboratory conditions for 7 days (S4–7 days).

In contrast, Figure 10 presents the gas evolution characteristics for core samples produced with the APNB binder system and stored for 7 days under controlled laboratory conditions (S4, T = 20–21 °C, RH = 45–50%). These samples demonstrated the lowest total gas release of all examined variants. The extended storage time likely allowed for more complete cross-linking of the binder, which led to a lower concentration of residual binder components (resin and hardener) involved in the thermal decomposition process during casting. These findings highlight the key role of appropriate seasoning time and stable storage conditions in stabilizing the casting process. Extending the storage period under controlled environmental conditions can significantly reduce gas emissions, particularly from cores, thereby decreasing the likelihood of gaseous casting defects. The results are particularly relevant for cores with complex geometry or limited venting capacity, where effective gas evacuation is difficult.

**Figure 11:**
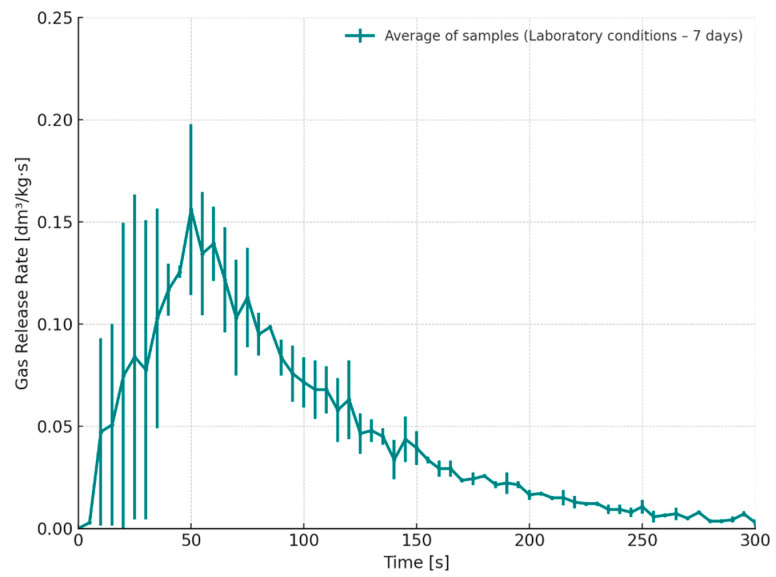
Gas release rate as a function of time for cores stored under laboratory conditions (S4–7 days).

Figure 11 presents the gas emission rate for core samples stored under laboratory conditions for 7 days (S4). These samples exhibited the lowest gas evolution rates among all tested variants. This observation suggests that extended storage under stable temperature and humidity conditions facilitates further chemical curing of the binder system, potentially leading to the formation of a more cross-linked structure that limits gas evolution during thermal exposure. As a result, the amount of gas released during contact with molten metal is significantly reduced. Additionally, the extended curing period contributes to a notable decrease in the peak emission rate, which reached approximately 0.18 dm^3^/kg·s on average. These values are considerably lower than those observed for cores subjected to thermal loading just 3 h after preparation, where incomplete curing may have contributed to more rapid and intense gas evolution.

**Figure 12:**
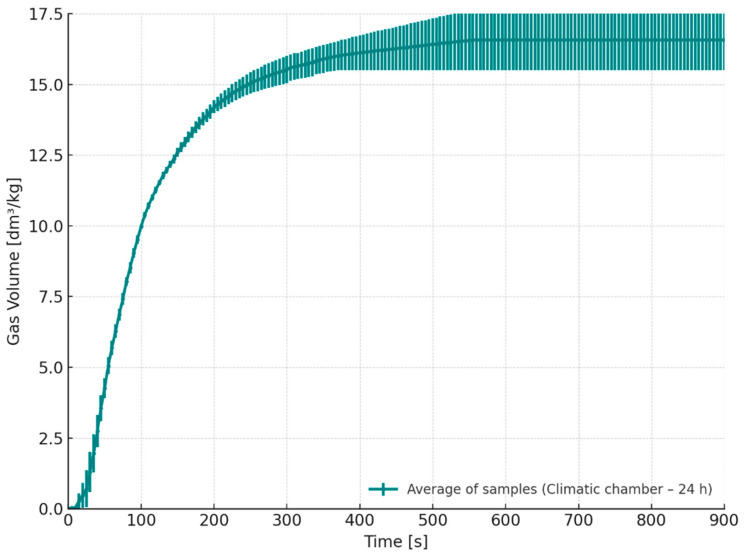
Released gas volume as a function of time for cores stored for 24 h in a climatic chamber (S5, temperature 35 °C, 70% humidity).

Increase in gas release can be attributed to the hygroscopic nature of resol-type phenol-formaldehyde resins, which readily absorb moisture from the environment under elevated humidity conditions. The absorbed water can interfere with the polycondensation reactions responsible for the curing process, as well as promote hydrolysis or slow down the cross-linking of the resin [25,26]. These effects may lead to alterations in the chemical structure of the binder, which in turn affect the kinetics of thermal decomposition when in contact with molten metal and influence the total volume of gas generated. This is confirmed by the data presented in Figure 12 (S5, climatic chamber—35 °C, 70% RH), showing that gas emissions from samples stored at 35 °C and 70% relative humidity are, on average, approximately 1 dm^3^/kg higher than those from samples stored under standard laboratory conditions.

**Figure 13:**
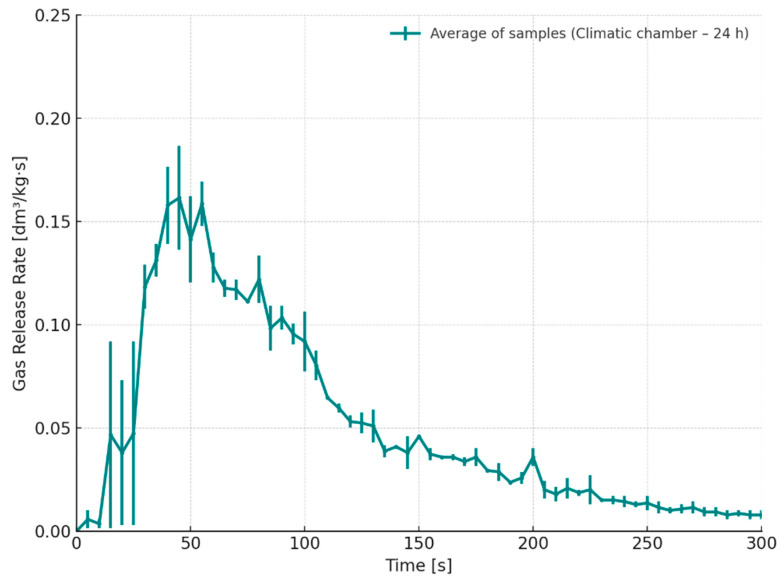
Gas release rate as a function of time for cores stored for 24 h in a climatic chamber (S5, temperature 35 °C, 70% humidity).

The gas emission rate from samples stored in the climate chamber exhibits a distinct peak during the initial phase of the process (Figure 13). For Sample I, the maximum emission rate occurred approximately 45 s after pouring, while for Sample J, it was observed around 55 s, yielding an average of 50 s post-pouring. It should be noted that, despite the increased total volume of gas generated, the rate of thermal decomposition product release is lower in this case; however, the emission process persists over a longer duration. This behavior may be associated with the previously mentioned changes in the chemical structure of the binder resulting from moisture exposure.

Table 3 presents a summary of the results of determining the volume of emitted gases over time for all tested samples, calculated per sample of known mass and per 1 kg of moulding/core sand, depending on the storage conditions.

Table 4 summarizes the mean values and standard deviations of the total gas volume and the maximum gas release rate for all storage variants (S1–S5). These results complement the data presented in Table 3 and provide a statistical comparison of gas-forming tendencies under different storage conditions.

Table 5 summarizes the results of BTEX compound determinations based on a sample of known mass, while Table 6 summarizes the results based on 1 kg of moulding/core sand.

The results of the BTEX compound measurements (benzene, toluene, ethylbenzene, xylenes) indicate a strong correlation between the storage conditions of the samples (cores) and the emission of benzene, the most hazardous component classified as a carcinogenic agent. Storing the cores in a desiccator significantly reduces benzene formation compared to storage under standard laboratory conditions. Seasoning the cores for 7 days has a beneficial effect on lowering BTEX emissions; for instance, benzene emissions were 14% lower compared to samples tested 3 h after preparation, and as much as 24% lower compared to those stored under laboratory conditions for 24 h. These findings suggest that storage conditions have a greater impact on benzene emission than the elapsed time between core preparation and casting. Notably, storage under elevated humidity conditions (climate chamber: T = 35 °C, RH = 70%) leads to a significant increase in BTEX emissions. In fact, benzene release increased by approximately 45% compared to cores seasoned for 7 days under standard laboratory conditions (T = 20–21 °C, RH = 45–50%).

## 4. Conclusions

Based on the study, the following conclusions were made:

- Storing samples in a desiccator for 24 h results in lower emissions compared to samples stored in the laboratory for the same period. In contrast, samples (cores) poured 3 h after preparation exhibit higher emissions than those poured after 24 h of storage, which is due to the evaporation of volatile substances from the hardener and/or the complete reaction of the binder components (resin–hardener).
- It has been demonstrated that storing samples under conditions of elevated humidity (temperature: 35 °C, humidity: 70%) leads to higher emissions, although still at a lower level than in samples poured 3 h after preparation. Therefore, it can be concluded that the “setting time” of the cores plays a crucial role in foundry practice, as it can significantly affect the tendency to form gas-related defects. Such defects are often irreparable and result in castings being classified as non-compliant with customer requirements. In many cases, these defects are discovered during machining or, even more critically, by the end user, resulting in high financial and reputational costs.
- Seasoning of cores for an adequate duration under controlled climatic conditions helps reduce both the overall gas generation and the emission rate. This is particularly important for cores exposed to extreme thermal loads, which are typically completely burned through during casting, causing total degradation of the binder and release of volatile decomposition products. Gas evacuation from such cores is limited, as they are almost entirely surrounded by molten metal. Therefore, any increase in gas volume generated during mould or core pouring becomes critically important and must be effectively vented to minimize the risk of gas-related defects.
- A properly designed core production process, including a sufficiently long seasoning period in controlled temperature and humidity conditions, can significantly reduce the risk of casting defects, improve product quality, and enhance occupational safety in foundries.
- The intensification of gas emissions under adverse storage conditions, especially high humidity, in APNB binder technology cores promotes the formation of toxic compounds from the BTEX group (benzene, toluene, ethylbenzene, xylenes). This not only leads to increased environmental emissions but also directly exposes foundry workers to harmful substances. Benzene is of particular concern, as it is classified as a carcinogenic compound.

## Figures and Tables

**Table 1:** Grain matrix parameters.

Parameter	Symbol	Value	Unit
Major fraction	F	0.32/0.20/0.16	mm
AFS particle number	L	56.10	-
Average particle size	d_L_	0.23	mm
Geometric mean	d_g_	0.25	mm
Arithmetic mean	d_a_	0.26	mm
Harmonic mean	d_h_	0.24	mm
Median	d_M_	0.25	mm
Average particle size	D50	0.25	mm
Major fraction	F_g_	87.25	%
Distribution coefficient	S_0_	1.22	-
Slope index	S_k_	0.96	-
Degree of homogeneity	GG	75.00	%
Specific surface area	S_t_	9.59	m^2^/kg

**Table 2:** Summary of experimental series used in the research.

Experimental Series	Storage Environment	Storage Duration
S1	Glass desiccator–dry conditions	24 h
S2	Laboratory conditions (20–21 °C, 45–50% RH)	3 h
S3	Laboratory conditions (20–21 °C, 45–50% RH)	24 h
S4	Laboratory conditions (20–21 °C, 45–50% RH)	7 days
S5	Climatic chamber (35 °C, 70% RH)	24 h

**Table 3:** Volume of gases from the tested samples in dm^3^/sample and dm^3^/1 kg of moulding/core sand (on the base [27,28]).

Experimental Series	Sample Description (Average of Samples)	Gas Volume [dm^3^/Sample]	Gas Volume [dm^3^/kg of Moulding Sand]
S1	24 h desiccator	2.16	15.80
S2	3 h laboratory	2.64	19.00
S3	24 h laboratory	2.20	15.95
S4	7 days laboratory	2.02	14.39
S5	24 h climatic chamber *	2.32	16.57

* temperature—35 °C, humidity—70%.

**Table 4:** Mean values and standard deviations of the total gas volume and the maximum gas release rate for all storage variants (S1–S5).

Experimental Series	Storage Condition	Gas Volume [dm^3^/kg]	Gas Release Rate [dm^3^/(kg·s)]
S1	24 h desiccator	15.83 ± 0.35	0.22 ± 0.01
S2	3 h laboratory	19.00 ± 0.70	0.32 ± 0.10
S3	24 h laboratory	16.09 ± 0.32	0.21 ± 0.00
S4	7 days laboratory	14.39 ± 0.28	0.18 ± 0.02
S5	24 h climatic chamber *	16.57 ± 1.50	0.17 ± 0.03

* temperature—35 °C, humidity—70%.

**Table 5:** BTEX emissions from moulding/core sand (mg/sample). Development of results based on [27,28].

Experimental Series	Sample Description (Average of Samples)	Gas [mg/Sample]			
		Benzene	Toluene	Ethylbenzene	Xylenes
S1	24 h desiccator	52.13	0.22	0.00	0.61
S2	3 h laboratory	63.66	3.36	0.00	2.19
S3	24 h laboratory	70.36	2.12	0.00	0.72
S4	7 days laboratory	56.14	0.57	0.00	1.24
S5	24 h climatic chamber *	80.98	1.26	0.00	0.96

* temperature—35 °C, humidity—70%.

**Table 6:** BTEX emissions from moulding/core sand (mg/1 kg moulding/core sand). Development of results based on [27,28].

Experimental Series	Sample Description (Average of Samples)	Mass of the Sample [g]	Gas [mg/kg of Moulding/Core Sand]			
			Benzene	Toluene	Ethylbenzene	Xylenes
S1	24 h desiccator	136.51	328.18	1.65	0.00	4.45
S2	3 h laboratory	139.52	455.85	2.98	0.00	15.66
S3	24 h laboratory	141.96	495.67	14.91	0.00	5.05
S4	7 days laboratory	140.17	400.36	4.06	0.00	8.83
S5	24 h climatic chamber *	140.13	577.97	9.03	0.00	7.02

* temperature—35 °C, humidity—70%.

## Data Availability

The original contributions presented in this study are included in the article. Further inquiries can be directed to the corresponding author.
